# Popliteal lymphadenectomy for treating metastatic melanoma: case report

**DOI:** 10.1590/S1516-31802008000400009

**Published:** 2008-07-03

**Authors:** Sergio Renato Pais Costa, Sergio Henrique Couto Horta, Alexandre Cruz Henriques

**Keywords:** Melanoma, Lymphatic metastasis, Lymphadenectomy, Skin neoplasms, Lower extremity, Melanoma, Metástase linfática, Linfadenectomia, Neoplasias cutâneas, Extremidade inferior

## Abstract

**CONTEXT:**

Regional lymph node involvement in patients with malignant melanomas has been associated with poor prognosis. In-transit metastases also lead to poor long-term survival. Whereas for nodal disease only regional lymphadenectomy offers adequate locoregional control, for in-transit metastasis both local excision and isolated limb perfusion with chemotherapy plus tumor necrosis factor-alpha can be used for disease control. In cases of tumors located in the distal region of the legs, the lymphatic dissemination most commonly observed is to the inguinal chain. Consequently, therapeutic inguinal lymphadenectomy or even selective lymphadenectomy (sentinel lymph node biopsy) have been recommended. On the other hand, involvement of the popliteal chain is very rare. When this occurs, popliteal lymphadenectomy should be indicated. Local excision may be the logical approach for a few small in-transit metastases because of the low morbidity in this procedure, when compared with isolated limb perfusion.

**CASE REPORT:**

A case of melanoma of the heel with popliteal chain involvement and in-transit metastases is presented. This was treated by means of regional lymphadenectomy plus in-transit metastases excision, with a good postoperative course.

## RESUMO

**CONTEXTO:**

O acometimento de linfonodos regionais em pacientes portadores de melanoma maligno tem sido associado a um prognóstico sombrio. Paralelamente, metástases em trânsito são também consideradas de mau prognóstico. Enquanto que para as metástases linfonodais apenas a linfadenectomia regional oferece um controle loco-regional adequado, para as metástases em trânsito por sua vez tanto a excisão local quanto a perfusão isolada de membro com quimioterapia e fator de necrose tumoral alfa podem ser utilizadas para seu tratamento. Nos tumores localizados na região distal dos membros inferiores, a disseminação linfática mais comumente observada é para cadeia inguinal. Conseqüentemente, a linfadenectomia inguinal terapêutica ou mesmo a linfadenectomia seletiva (biópsia de linfonodo sentinela) têm sido recomendadas. Em contrapartida, o acometimento da cadeia poplítea é muito raro. Quando ocorre, a linfadenectomia poplítea deve ser indicada. Concomitantemente, na vigência de um número pequeno de metástases em trânsito, a excisão local é uma alternativa interessante haja vista sua menor morbidade quando comparada a perfusão isolada do membro.

**RELATO DE CASO:**

Os autores apresentam um caso de melanoma de calcanhar com acometimento da cadeia poplítea e metástases em trânsito que foi submetido à linfadenectomia regional e excisão das metástases em trânsito com boa evolução pós-operatória.

## INTRODUCTION

A variety of prognostic factors relating to survival have been reported for cases of malignant melanoma. However, among the factors most frequently described, the presence of distant metastases and the involvement of regional lymph nodes seem to be associated with poor prognosis.^1,2^ For melanomas located in the distal region of the legs, the usual location that is primarily affected by lymph node metastases is the inguinal chain. More rarely, the popliteal chain is the one involved. Although the latter situation is much less frequent in clinical practice, popliteal lymphadenectomy has been the procedure of choice for its treatment when present (in cases of localized disease).^3,4^ Likewise, it has also been recommended for cases in which there are few in-transit metastases.^2^

In the present study, a case of malignant melanoma in the leg (heel region) with primary involvement of the popliteal lymph node chain is described. This patient underwent widening of the primary tumor margin, together with resection of the in-transit metastases and popliteal lymphadenectomy, in a single operation.

## CASE REPORT

The patient was a 65-year-old white woman who was referred to the Oncology Service following excisional biopsy of a lesion in the left heel region one month earlier. Histological examination had revealed a malignant melanoma that presented Breslow classification of 3 mm with Clark IV. On physical examination, she not only presented a hardened lesion in the left heel of 2 cm in diameter, but also three other lesions (with the same characteristics and dimensions) close to it, in the calf region. Palpation of the popliteal fossa showed that there were two lymph nodes with hardened consistency (mobile in relation to deep structures), of approximately 2.5 cm in diameter. The inguinal region did not present any abnormalities upon physical examination. In the light of this clinical picture, the diagnostic hypothesis was malignant melanoma with in-transit metastases and involvement of the popliteal lymph node chain.

Subsequently, computed tomography scans of the patient's legs, pelvis, abdomen and chest were undertaken to stage the disease. These images did not produce any evidence of distant dissemination of the disease. Therefore, resection of the in-transit metastases together with popliteal lymphadenectomy with curative intent was indicated.

**Figure 1:**
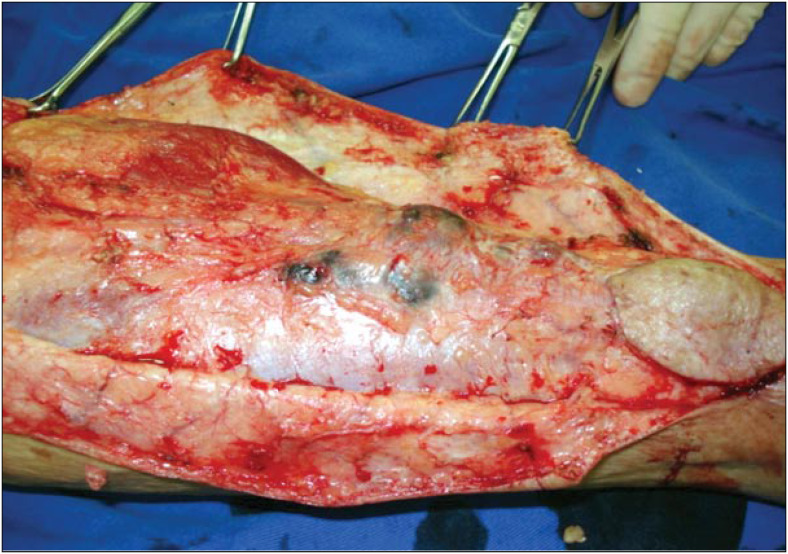
Resection of the in-transit metastases.

**Figure 2:**
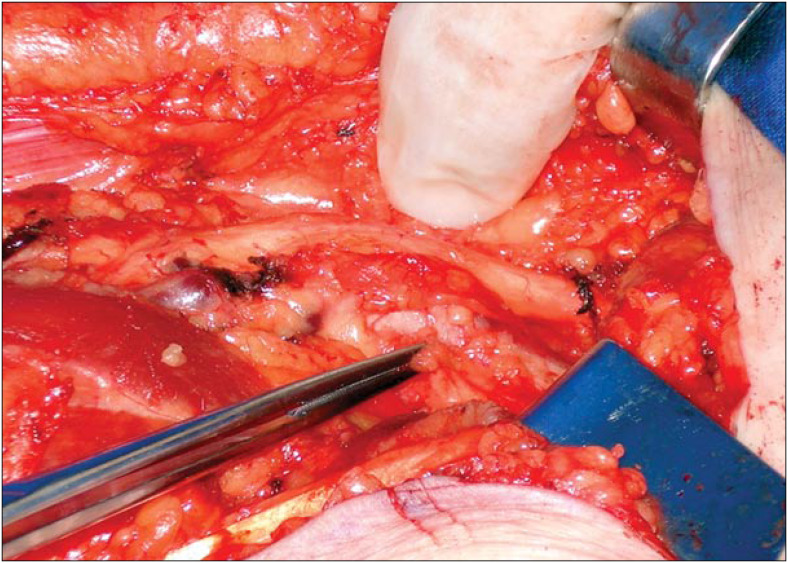
Popliteal lymphadenectomy.

The access route was an S-shaped incision (with zetaplasty) along the left leg, going from the heel region to the popliteal cavum. At the same time as widening the margin of the primary lesion by two centimeters, three in-transit metastases were resected (Figure 1) and monoblock popliteal lymphadenectomy was performed (Figure 2), in accordance with the technique described by Karakousis.^5^ The patient's course did not present complications, and she was discharged on the fifth postoperative day.

Histological examination of the operative specimen showed absence of any tumor in the primary lesion and presence of metastases in all seven of the lymph nodes dissected. We preferred to perform the surgical procedure in two stages since the patient presented several associated diseases. Consequently, one month after this operation, the patient underwent complementary inguinal lymphadenectomy, which followed an uneventful course, and no metastases were found in the 15 lymph nodes dissected. Up to the present time (four months after the popliteal lymphadenectomy), the patient has not presented any signs of clinical or radiological recurrence.

## DISCUSSION

Lymphatic metastases to the popliteal chain from melanomas located in the distal region of the lower limbs (lower legs or feet) are extremely rare. In general, tumors in this location more frequently present lymphatic dissemination to the inguinal region. Moreover, since the lymph nodes of this chain are located deeply in relation to the muscle fascia, palpation of these nodes in the physical examination is very problematic.^3^

This propaedeutic obstacle tends to lead to delayed diagnosis, which therefore makes it possible that the disease will only be discovered at a late stage. Distant metastases are invariably already present when this lymph node chain is involved.^6^

Two drainage routes have usually been described for melanomas of the legs: a principal route that originates from the medial portion of the foot and drains towards the inguinal region, and a secondary route that originates from the lateral region of the foot and drains towards the popliteal region.^7^ On the other hand, it has been suggested that tumors located in the posterolateral region of the heel below the lateral malleolus, as observed in the present case, have preponderant dissemination to the popliteal region.^4^ Thompson et al.^8^ also demonstrated that any melanoma below the knee could present dissemination to the lymph nodes along the popliteal fossa.

Lymphatic dissemination to the popliteal chain may occur concomitantly to dissemination to the inguinal chain, or even subsequently (“backflow”). In a less frequent form, this phenomenon may possibly be associated with in-transit metastases, as in the present case.^6,9^

In-transit metastases occur in approximately 5% to 8% of patients with high-risk melanoma. The management of in-transit metastases remains a challenge because it is dictated by the biological behavior of melanoma, especially in terms of the number and size of the lesions. Surgical excision of in-transit metastases is chosen when the size and number of the lesions allow this approach, as in the present case. It must be noted that amputation is seldom if ever indicated and does not improve survival. The excision should be radical, but no specific excisional margin has been proven to be beneficial for these metastases, contrary to cases of primary melanoma. The possibility of repeated excision is completely dictated by the location, the size and, for practical reasons, the number of the lesions.^10,11^

The access route that has been recommended^12^ for removing popliteal material is a large S-shaped incision (zetaplasty), as in the case reported here. This approach has the main objectives of reducing possible scar retraction and obtaining excellent exposure of the popliteal cavum with its neurovascular structures. The initial step in the dissection is to obtain adequate exposure of the popliteal fossa, with identification of the neurovascular bundle. Identification of the popliteal fascia, which is extremely thin and friable, is the basic step in the operation. This structure serves as the main anatomical reference point because of its proximity to the neurovascular bundle. When the fascia has been exposed, the more superficial structures like the short saphenous vein and some superficial nerves are dissected and ligated. Care must be taken close to the deep portion of the fascia, because the nerves are close to the surface. The tibial nerve is the most superficial structure of the fossa, and the popliteal vessels are located below it. A portion of weak areolar tissue is seen along the popliteal vessels, and this includes between two and seven lymph nodes. With careful dissection, this weak areolar tissue is removed together with the lymph nodes along the neurovascular bundle.^12^

When numerous small lesions exist, local ablation by means of carbon dioxide laser therapy may be useful, since it minimizes the injury to surrounding tissue and enables treatment of multiple lesions in a single session. The drawback of this is that the healing by secondary intention may be lengthy and painful. Although the initial results have been reported to be adequate, the recurrence rate may be very high. This, together with the inability of this technique to treat lesions greater than 1 cm in diameter, has limited the use of carbon dioxide laser ablation to a very specific patient population.^10^

Although melanoma cells are relatively resistant to radiation, radiotherapy can be used for local control treatment in cases of cutaneous melanoma, at sites that would otherwise require complex surgical procedures. In cases of gross disease in a relatively small total area, radiotherapy has proven to be effective in providing local control (in approximately 50% of such cases). Its effectiveness might even be increased by adding the use of hyperthermia techniques in centers with appropriate expertise and equipment.^10^

Apart from local control, melanoma is refractory to virtually all systemic treatments. Therefore, when multiple in-transit metastases occur, various locoregional approaches have been proposed and investigated. Isolated limb perfusion (ILP), which was developed by Creech et al. in 1958,^13^ is the most effective regional treatment method, because it achieves tissue concentrations of the chemotherapeutic agents in the affected limb that are more than 20 times higher than what can be achieved systemically.^14^ Melphalan (L-phenylalanine mustard [L-PAM], Alkeran^®^, Wellcome, London, United Kingdom) has been used as the standard drug for ILP over the years because of its efficacy and low toxicity.^15^ Hyperthermia may increase the response rates somewhat, but at the cost of locoregional toxicity. Melphalan-based ILP for in-transit melanoma metastases is associated with complete response rates of 40% to 50% and overall response rates of 75% to 80%.^16^ Large melanoma lesions are difficult to eradicate because of poor and non-homogeneous drug uptake, as in the case of soft-tissue sarcomas. Therefore, ILP programs using melphalan alone have been abandoned for treating unresectable soft-tissue sarcomas.^17^ The application of tumor necrosis factor-alpha (TNF)^18^ has changed this situation dramatically, because very large tumors are now seen to respond very well. Consequently, TNF has also been used increasingly in combination with melphalan for treating in-transit metastases by means of ILP. An early report on TNF-based ILP from four centers in Europe showed significantly increased complete response rates, of up to 90%, compared with a 52% complete response rate following ILP in these centers when melphalan alone was used.^19^ Finally, Grünhagen et al.^11^ demonstrated the very high efficacy of TNF-based ILP in melanoma patients, in terms of both local disease control and survival. The outcome is influenced by the disease stage, thus reflecting the aggressiveness of the melanoma. According to these authors, TNF-based ILP should be considered in all cases of limb-threatening tumors or in situations where simple surgical procedures to obtain local control fail.

With the advent of sentinel lymph node investigations (gamma probes or vital staining), together with routine use of lymph scintigraphy, there has been an increase in the frequency of diagnosing this lymph node chain. Whereas rarely reported prior to these technological innovations, popliteal involvement has now been reported in greater percentages that range from 1% to 20% of all melanomas of the legs.^9^

Despite this greater observed prevalence, involvement of the popliteal chain still remains unusual. According to Marone et al.,^4^ out of 148 patients with melanoma of the legs who underwent sentinel lymph node investigation between 1996 and 2005, primary drainage to the popliteal chain was only observed on lymph scintigraphy in two cases (1.3%). Moreover, in both of these cases, no metastases were observed in the histological evaluation of the sentinel lymph nodes that were resected. In this same sample, clinical metastatic dissemination to the popliteal chain was only observed in one case (only 0.7% of all the cases studied).

Thompson et al.^8^ conducted a retrospective study on 4,262 patients with melanoma of the legs, among whom only 13 patients (0.3%) presented lymph node metastases to the popliteal chain. Five of these 13 patients presented involvement of the inguinal chain concomitantly or prior to the involvement of the popliteal chain. Eight of these patients with metastases in the popliteal chain subsequently developed systemic disease over a mean time period of 39 months (ranging from three to 127 months) after their primary treatment. At the time of the last reported follow-up (median of 97 months), seven patients had already died due to their melanoma. Only six of the patients were still alive and continued to be free of disease over this follow-up period. These authors concluded that if lymph nodes in the popliteal fossa were detected by lymph scintigraphy, sentinel lymph node biopsy should be indicated. Furthermore, if neoplasm were to be histologically confirmed, popliteal lymphadenectomy should be performed with therapeutic intent.

Menes et al.^9^ reported that, out of a group of 106 patients with melanoma of the leg or foot, ten patients (9%) showed positive drainage to the popliteal fossa on lymph scintigraphy. Thus, they identified a metastatic involvement rate of 2.8% for the popliteal chain. Lymph node metastases were identified by immunohistochemistry in only one patient, while in another two patients the lymph nodes involved were already clinically palpable at the time of diagnosis. All these patients presented concomitant drainage to the inguinal region that was clinically evident or was seen by means of lymph scintigraphy. Even though all these three patients were adequately treated from an oncological point of view (selective popliteal lymphadenectomy in one patient and popliteal and inguinal lymphadenectomy required in two patients), they presented recurrence within a short space of time (three months). All these patients developed systemic disease and in-transit metastases and finally died of the disease. These authors concluded that the popliteal fossa should be systematically examined in patients with melanomas located in the distal region of the legs. In addition, even if exclusively popliteal metastatic disease were observed, popliteal lymphadenectomy should be the standard therapeutic procedure followed.

## CONCLUSION

The present case described a peculiar dissemination of melanoma of the legs that is rarely seen in daily practice. However, this particular presentation is resolved by a therapeutic approach that is safe and effective despite only being used exceptionally.
